# Supplementary material for: Leucine-rich repeat receptor-like gene screen reveals that Nicotiana RXEG1 regulates glycoside hydrolase 12 MAMP detection
Source: Nat Commun. 2018 Feb 9;9:594. doi: 10.1038/s41467-018-03010-8 (PMC5807360; doi:10.1038/s41467-018-03010-8)
Supplement: Supplementary file 1 — Supplementary Information [file 41467_2018_3010_MOESM1_ESM.pdf]

Supplementary Information

Leucine-rich repeat receptor-like gene screen reveals that *Nicotiana* RXEG1 regulates glycoside hydrolase 12 MAMP detection

Wang et al.

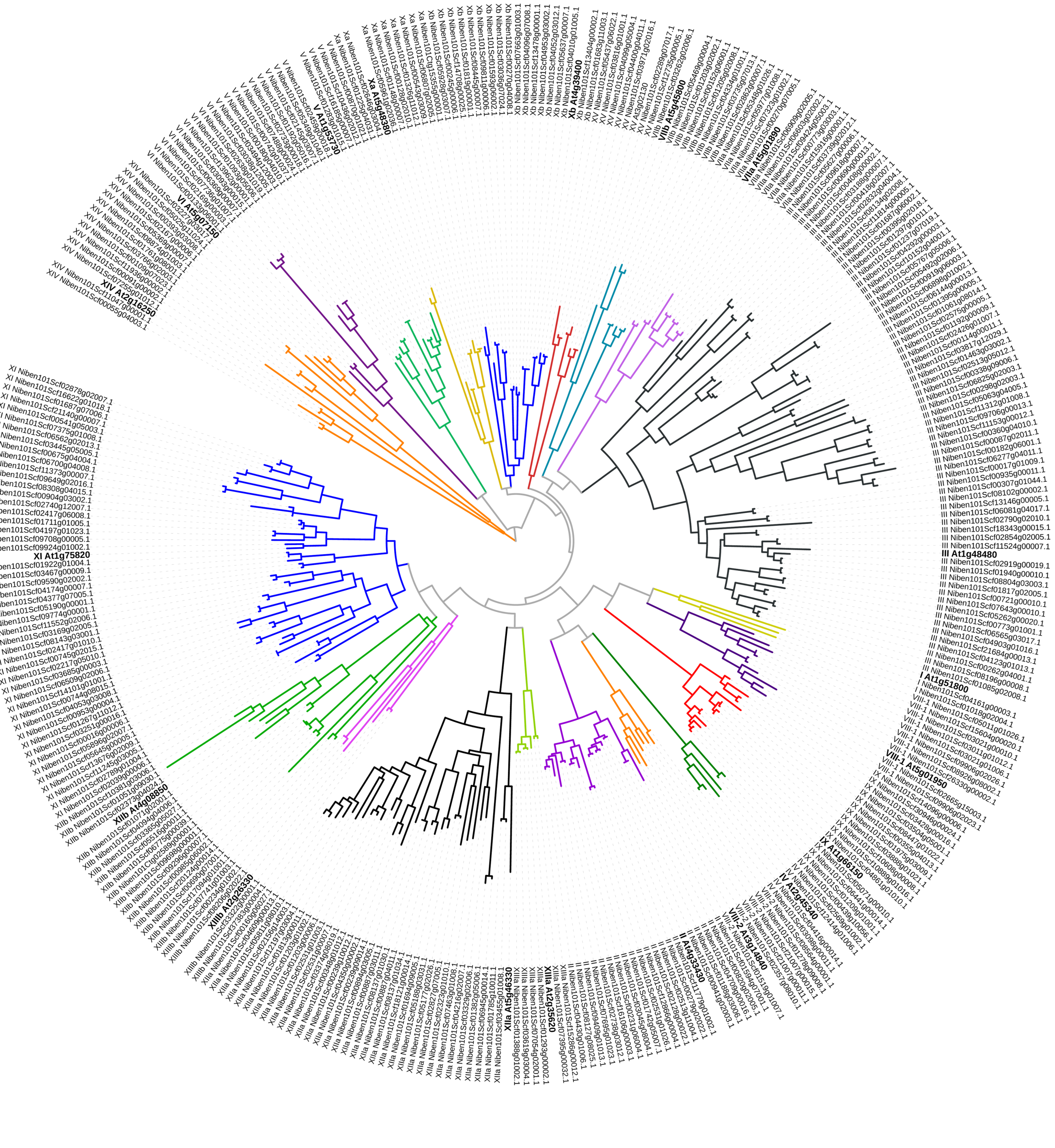

Supplementary Figure 1. Phylogenetic analysis of LRR-RLKs in *Nicotiana benthamiana*. LRR-RLKs in *Arabidopsis thaliana*, which represent the 20 subgroups[1,2], were used as references. Different colors indicate different subgroups. Phylogenetic maximum-likelihood (ML) tree was constructed using the predicted kinase domain sequences and PhyML implemented in SEAVIEW software (http://doua.prabi.fr/software/seaview). The phylogenetic trees were displayed using iTOL (http://itol.embl.de).

a

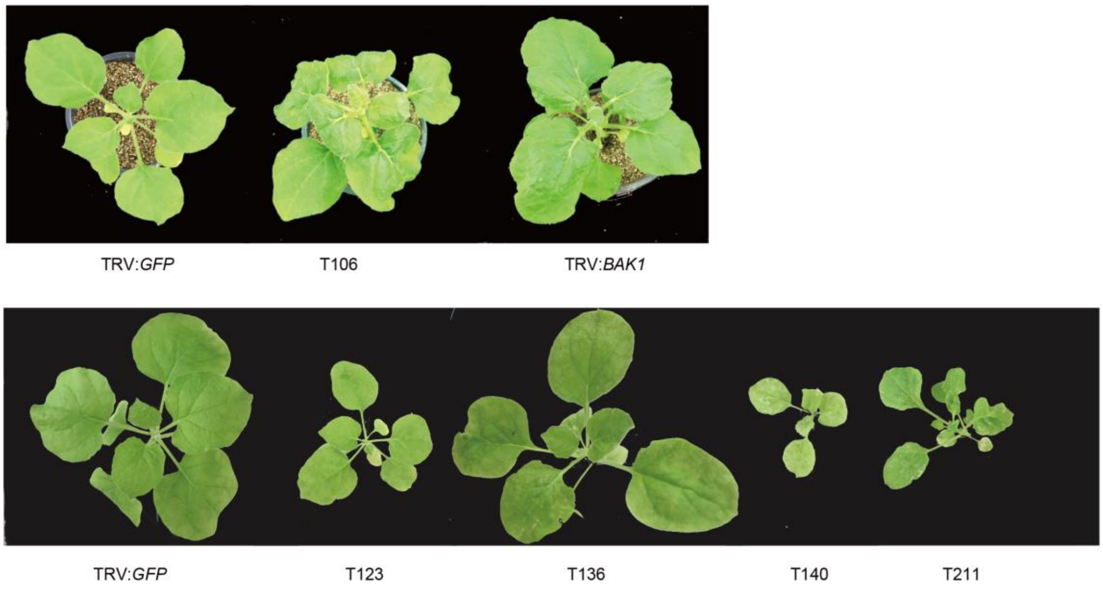

b

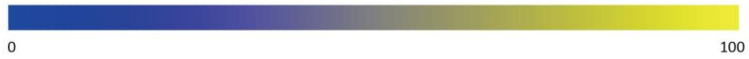

Construct	Type	Targets	No. of LRRs	Sequence Similarity				
				S. lycopersicum	*S. tuberosum*	*C. annuum*	*G. max*	*A. thaliana*
T106	RD-LRR-RLK	Niben101Scf01683g11003.1	21	88.58	88.82	88.23	69.86	66.69
	RD-LRR-RLK	Niben101Scf13404g00002.1	22	88.58	88.73	88.31	69.96	66.61
T123	GH-LRR-RLK	Niben101Scf11153g00012.1	5	77.96	78.85	76.61	48.3	50.23
	LRR-RLP	Niben101Scf02298g01015.1	5	76.1	44.5	63.4	49.5	30.8
T136	RD-LRR-RLK	Niben101Scf10608g00008.1	9	78.06	87.91	86.38	65.73	61.41
	RD-LRR-RLK	Niben101Scf03888g01021.1	9	78.05	87.15	85.27	65.13	60.71
T140	GN-LRR-RLK	Niben101Scf04292g00003.1	9	77.6	77.59	68.02	67.2	61.6
	GN-LRR-RLK	Niben101Scf10152g04001.1	9	77.85	79.59	68.53	67.88	62.81
T211	GN-LRR-RLK	Niben101Scf06204g00001.1	3	73.68	75.53	76.4	46.55	32.18
	LRR-RLP	Niben101Scf00683g02006.1	4	71.8	12.6	79.2	43.2	23.5

Supplementary Figure 2. Several conserved LRR-RLKs play a potential role in plant development.

a, Morphology of TRV construct-treated *N*. *benthamiana* plants. Plants were photographed five weeks after silencing.

b, Conservation of LRR receptor-like genes with a potential role in plant development among different plant species. The number indicates the similarity with their orthologs in indicated plant species.

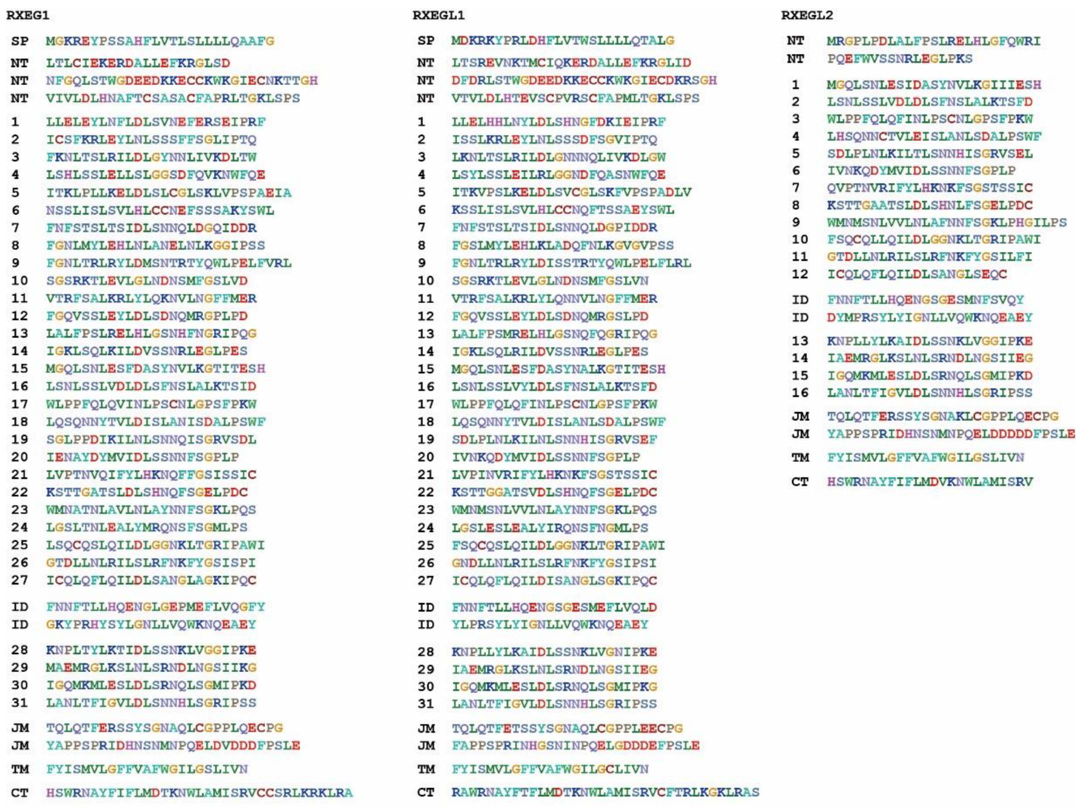

Supplementary Figure 3. The domain compositions of RXEG1, RXEGL1 and RXEGL2. The LRRs were aligned and determined according to Jehle et al. 2013[3] and Zipfel et al. 2006[4]. SP, signal peptide; NT, N-terminus of the LRR domain; ID, island domain; JM, juxtamembrane domain; TM, transmembrane domain; CT, cytoplasmic tail.

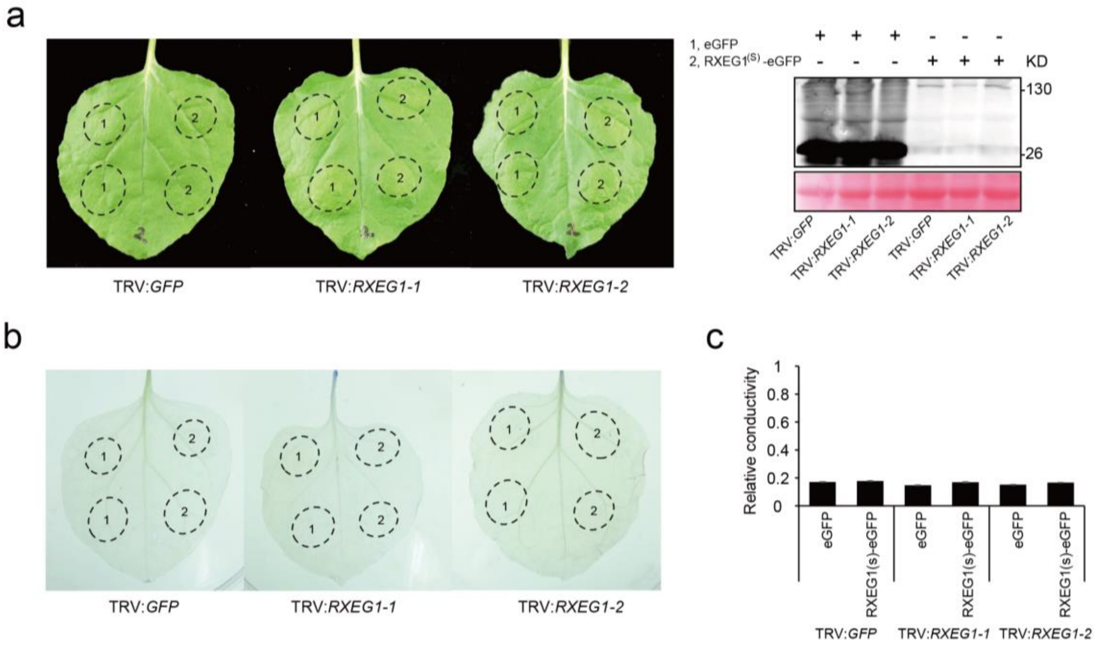

Supplementary Figure 4. Expression of RXEG1$^{(S)}$ in *Nicotiana benthamiana* does not induce plant cell death.

a, *Nicotiana benthamiana* leaves (n=6) expressing RXEG1$^{(S)}$-eGFP or eGFP photographed 3 days after infiltration (dai). RXEG1$^{(S)}$-eGFP or eGFP was co-expressed with P19 in *N*. *benthamiana* and protein accumulation was monitored 3 dai with anti-GFP antibody.

b, Representive *Nicotiana benthamiana* leaves expressing RXEG1$^{(S)}$-eGFP or eGFP stained with trypan blue.

c, Relative ion leakage in leaves expressing RXEG1$^{(S)}$ -eGFP or eGFP. Leaf discs were collected 3 dai. Bars represent average value (± s.e.m) of six replicates.

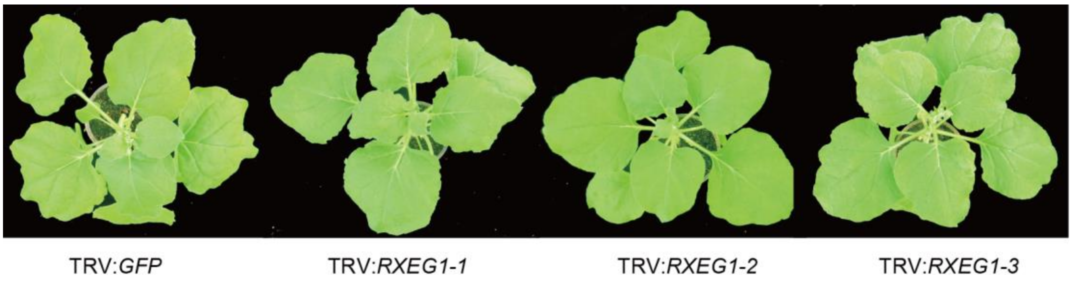

Supplementary Figure 5. Morphology of *N*. *benthamiana* plants treated with TRV:*GFP* or *RXEG1*-silencing constructs. Plants were photographed five weeks after silencing.

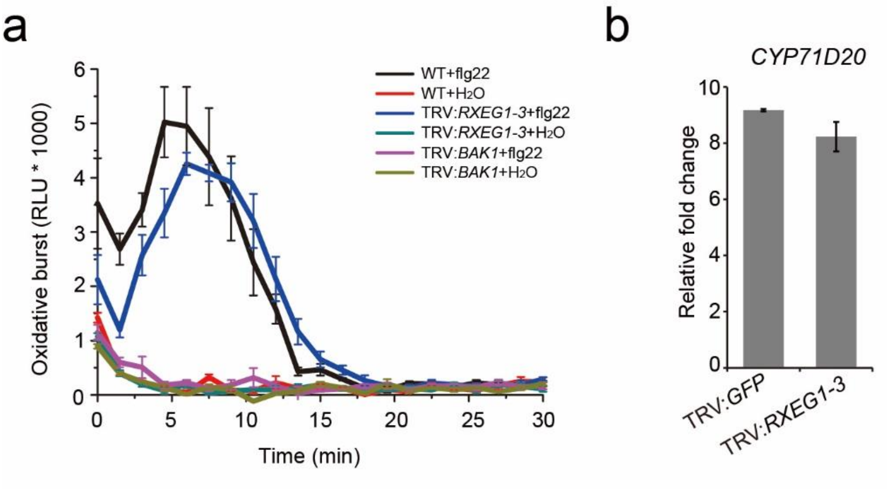

Supplementary Figure 6. Flg22-induced plant immune responses in TRV:*GFP* and TRV:*RXEG1-3*-treated *N. benthamiana*.

a, Production of ROS in *RXEG1*-silencing *N*. *benthamiana* treated with 100 nM flg22 or H_2O. Mean values (± s.e.m) of four replicates are shown. Experiment was repeated three times with similar results.

b, Relative expression of defense-related marker gene *CYP71D20* in *RXEG1*-silencing *N*. *benthamiana* leaves 3 h after treatment with 100 nM flg22 or H_2O. Transcript levels were quantified by qRT-PCR and normalized to EF-1α. Bars represent the mean fold changes (± s.e.m) of the flg22-treated leaves relative to the value in H_2O-treated leaves, which was set as 1. Experiment was repeated twice with similar results.

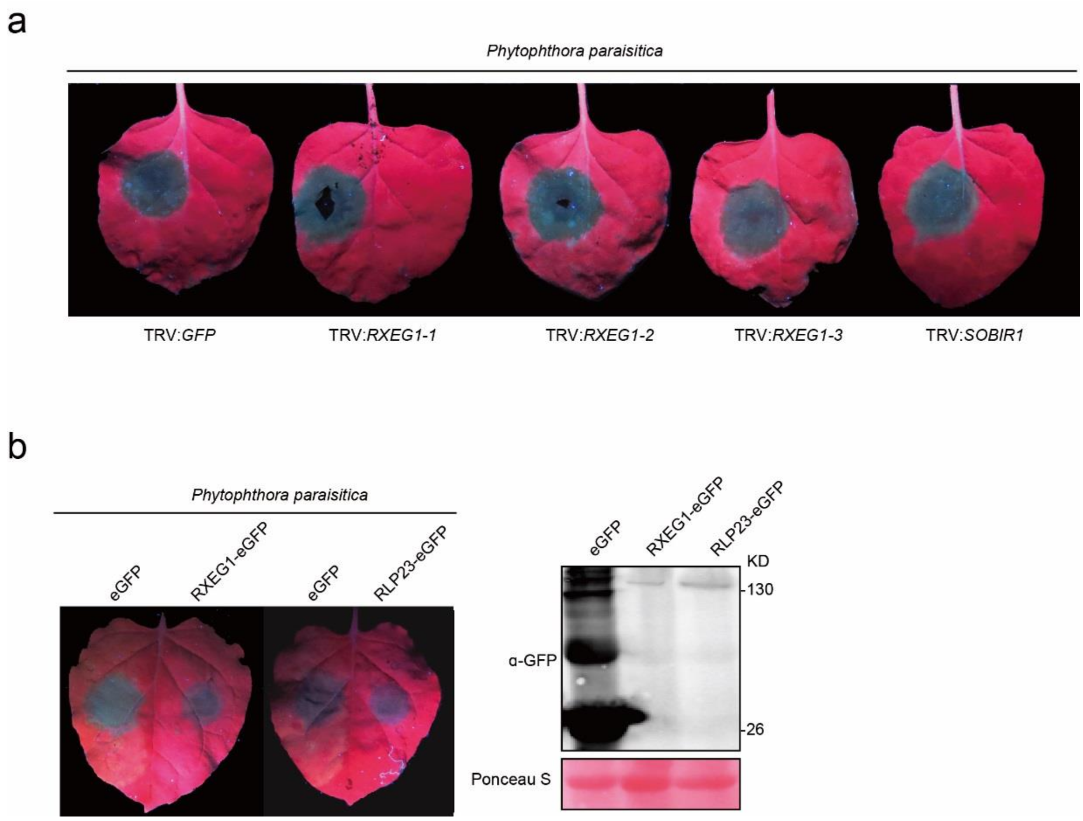

Supplementary Figure 7. Representative leaves showing the disease symptoms of *SOBIR1*-silencing, *RXEG1*-silencing (a) or overexpressing (b) *N. benthamiana* leaves upon infection with *Phytophthora parasitica*. *N. benthamiana* leaves (silencing, n=12; overexpressing, n=16) were inoculated with *P*. *parasitica* mycelial plugs and photographed at 3 days post inoculation (dpi). Experiments were repeated twice with similar results. Protein accumulation was monitored 2 dai with anti-GFP antibody. Protein loading is indicated by Ponceau S staining.

a

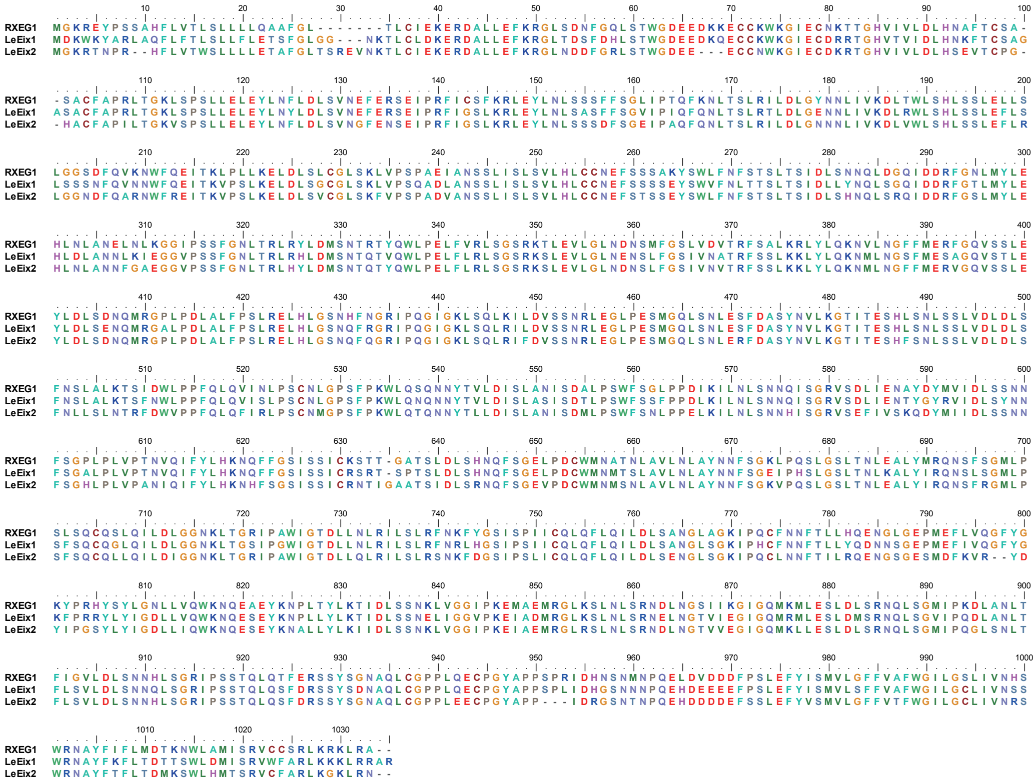

b

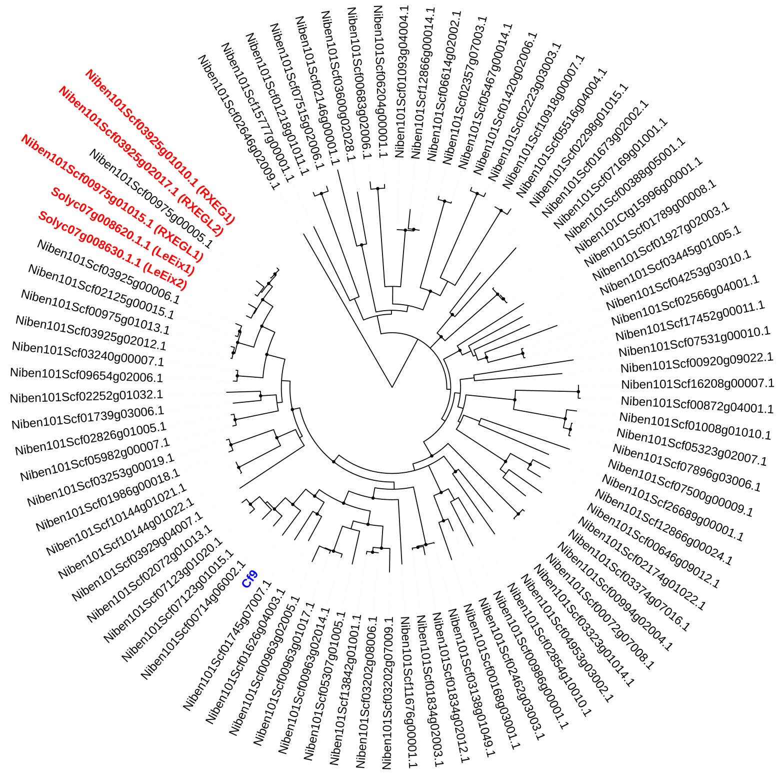

Supplementary Figure 8. Phylogenetic analysis of RXEG1 with homologs in *N. benthamiana* and tomato.

a, Sequence alignment of RXEG1 with LeEix1 and LeEix2 using ClustalW.

b, A phylogenetic tree was built using tomato LeEIX1/2 and all the identified *N. benthamiana* RLPs containing the C3-D domain[5] following the method described by Fritz-Laylin et al. 2005[6] and Petre et al. 2014[7]. The C3-D domain of tomato Cf9[5] was used as the reference.

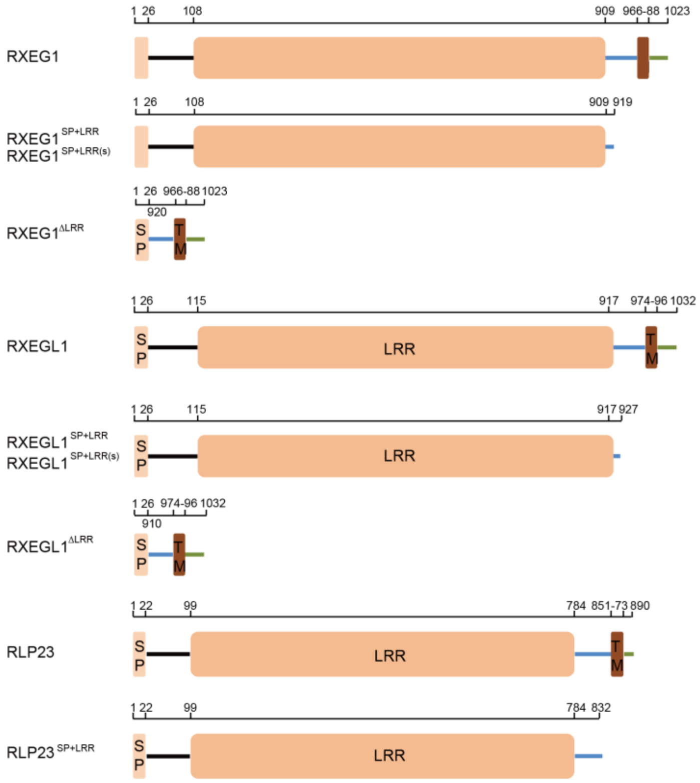

Supplementary Figure 9. Schematic representation of RXEG1, RXEGL1, *Arabidopsis* RLP23, and derivatives. Amino acids of each domain are indicated.

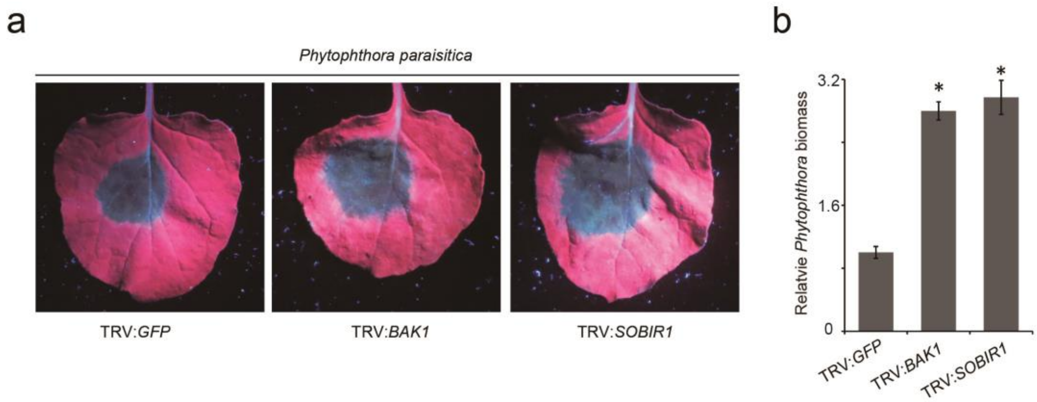

Supplementary Figure 10. Representative leaves showing the disease symptoms of *BAK1*-silencing and *SOBIR1*-silencing *N. benthamiana* leaves upon infection with *Phytophthora parasitica*.

a, *N. benthamiana* leaves (n=12) were inoculated with *P. parasitica* mycelial plugs and photographed at 3 dpi. Experiments were repeated twice with similar results.

b, Relative quantification of *P. parasitica* biomass in TRV:*GFP*, TRV:*BAK1* and TRV:*SOBIR1*-treated *N. benthamiana* leaves using qPCR. Infected leaves (n=12) were collected at 3 dpi and used for DNA isolation and qPCR analysis. *, significant differences ($P < 0.05$, Student's *t*-test). Experiments were repeated twice with similar results.

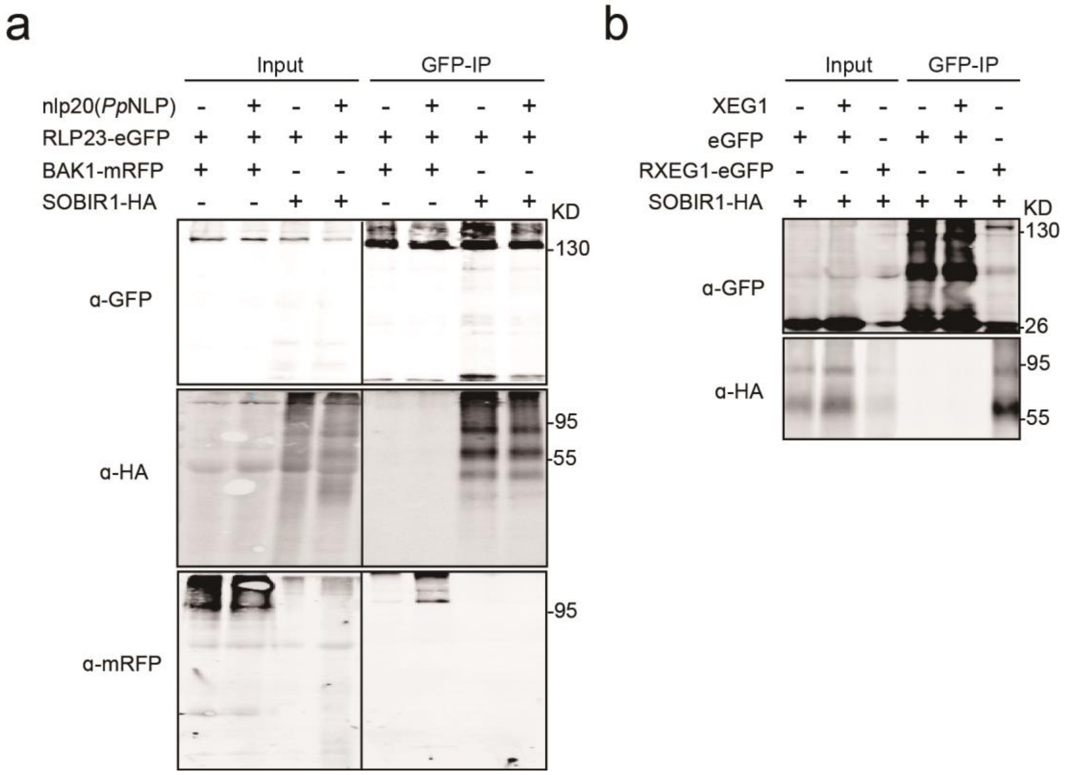

Supplementary Figure 11. Interactions of RLP23 or RXEG1 with BAK1 or SOBIR1 *in planta*.

a, RLP23 interacts with BAK1 in a nlp20-dependent manner while interacts constitutively with SOBIR1. *N. benthamiana* leaves were agro-infiltrated to express RLP23-eGFP, BAK1-mRFP, or SOBIR1-HA and collected 2 dai after treatment with 1 μM nlp20 (*Pp*NLP) or H_2O for 5 min. Extracted proteins were subjected to Co-IP using GFP-trap® A beads and western blotting with anti-GFP, anti-RFP, and anti-HA antibodies.

b, SOBIR1-HA interacts constitutively with RXEG1-eGFP but not with eGFP. SOBIR1-HA was co-expressed with RXEG1-eGFP or eGFP in *N. benthamiana* leaves. Infiltrated leaves were collected at 2 dai and treated with 1 μM XEG1 or EV for 10 min before protein isolation. Extracted proteins were subjected to Co-IP using GFP-trap® A beads and western blotting with anti-GFP and anti-HA antibodies.

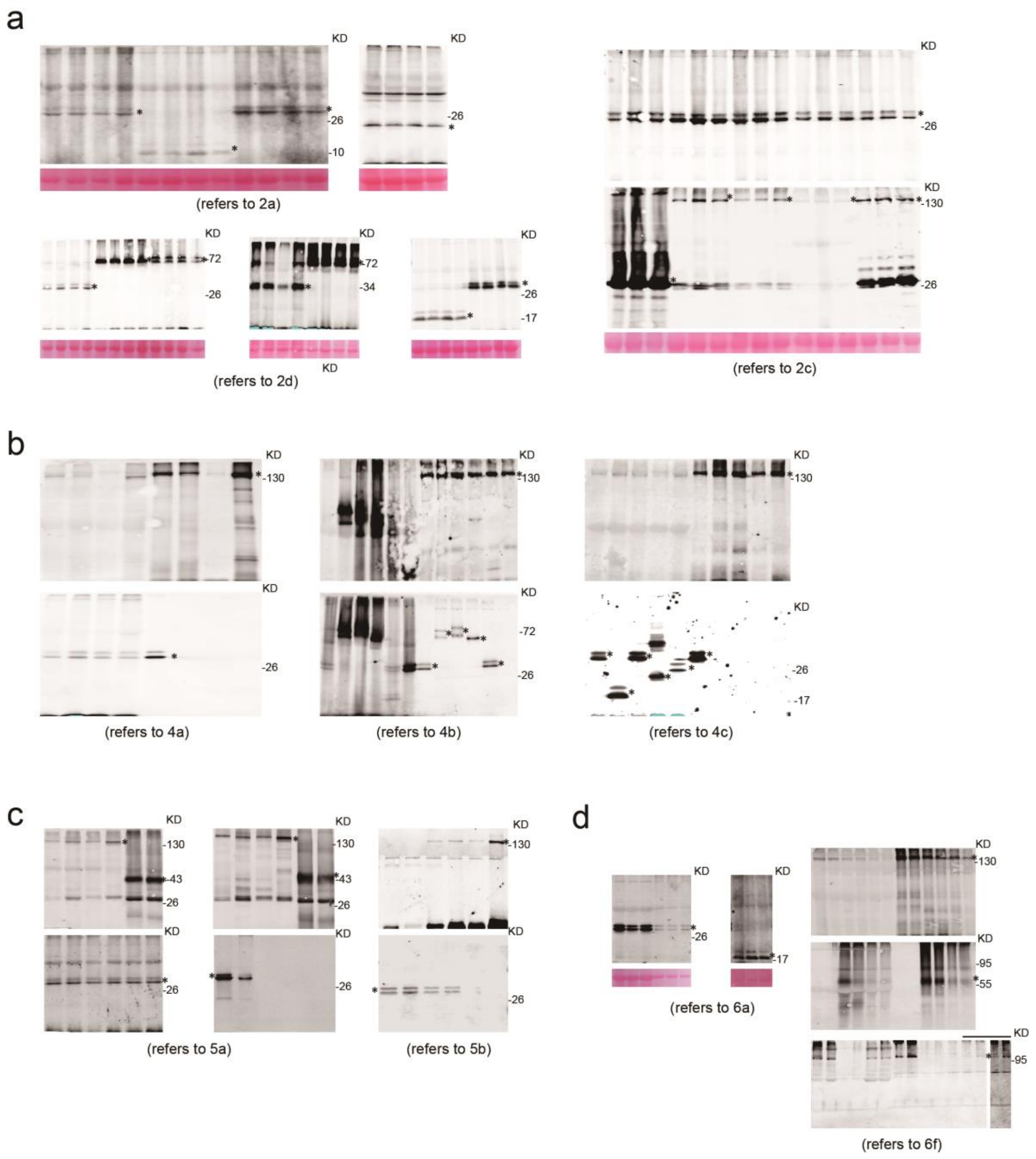

Supplementary Figure 12. Original western-blots shown in main Figure. * pointing to the band with expected sizes. – labeling the same blot visualized in different contrasts.

Supplementary Table 1. Primer list.

Primers	Sequences 5'-3'	Used for
pBin(c)GFP-RXEG1-F	TTACGAACGATAGCCGGTACCATGGGCAAAAGGGAATATCCAAG	cloning full length RXEG1 in *N. benthamiana*
pBin(c)GFP-RXEG1-R	GCTCACCATCCCGGGGGTACCAGCCCTTAACTTTCTCTTCAGTC	
pBin(c)GFP-RXEG1^{SP+LRR}-R	GCTCACCATCCCGGGGGTACCGGATGATCTCTCAAAAGTTTGG	cloning RXEG1^{SP+LRR} domain
pBin(c)GFP-RXEG1$^{SP+LRR(s)}$-R	GCTCACCATCCCGGGGGTACCCGATGACCTCTCAAATGTCTGG	cloning synthesized RXEG1^{SP+LRR} domain
pBin(c)GFP-RXEG1^{SP+TM}-R(F)	GCTTGATGGAATTCTCCCTGATCCAAAAGCTGCTTGTAGGAG	cloning RXEG1-ΔLRR domain
pBin(c)GFP-RXEG1$^{SP+TM\text{-}tail}$-F(R)	CTCCTACAAGCAGCTTTTGGATCAGGGAGAATTCCATCAAGC	cloning RXEG1-ΔLRR domain
pBin(c)GFP-RXEGL1-F	TTACGAACGATAGCCGGTACCATGGATAAAAGGAAATATCCAAG	cloning full length RXEGL1 in *N. benthamiana*
pBin(c)GFP-RXEGL1-R	GCTCACCATCCCGGGGGTACCTGAGGCCCTTAGCTTTCCCT	
pBin(c)GFP-RXEGL1^{SP+LRR}-R	GCTCACCATCCCGGGGGTACCGGATGATGTCTCAAAAGTTTGGAG	cloning RXEGL1-SP+LRR domain
pBin(c)GFP-RXEGL1$^{SP+LRR(s)}$-R	GCTCACCATCCCGGGGGTACCCGAAGATGTTTCAAATGTTTGC	cloning synthesized RXEGL1-SP+LRR domain
pBin(c)GFP-RXEGL1^{SP+TM}-R(F)	GCTTGATGGAATTCTCCCTGATCCTAAAGCTGTCTGTAAGAGC	cloning RXEGL1-ΔLRR domain
pBin(c)GFP-RXEGL1$^{TM\text{-}tail}$-F(R)	GCTCTTACAGACAGCTTTAGGATCAGGGAGAATTCCATCAAGC	cloning RXEGL1-ΔLRR domain
pBin(c)HA-SOBIR1-F	TTACGAACGATAGCCGGTACCATGGCCTTCACTGCTTCAC	cloning full length SOBIR1 in *N. benthamiana*
pBin(c)HA-SOBIR1-R	AACATCGTATGGGTACCCGGGATGCTTGATCTGAGTTAACATACACC	
pBin(c)mRFP-BAK1-F	TTACGAACGATAGCCGGTACCATGGATCAATGGCTATTGGGGATC	cloning full length BAK1 in *N. benthamiana*
pBin(c)mRFP-BAK1-R	AACATCGTATGGGTACCCGGGTCTTGGCCCTGATAACTCATC	
pBin(c)GFP-RXEGL2-F	TTACGAACGATAGCCGGTACCATGAGAGGGCCATTACCAG	cloning full length RXEGL2
pBin(c)GFP-RXEGL2-R	GCTCACCATCCCGGGGGTACCGACTCTTGATATCATAGCGAGC	
pBin(c)GFP-AtRLP23-F	TTACGAACGATAGCCGGTACCATGTCAAAGGCGCTTTTGCAT	cloning full length AtRLP23
pBin(c)eGFP-AtRLP23-R	GCTCACCATCCCGGGGGTACCACGCTTTCTGCGTTTATTCAG	
pBin(c)GFP-RLP23^{SP+LRR}-R	GCTCACCATCCCGGGGGTACCGTCTTCTTGCTTTTGGTGATAC	cloning AtRLP23-SP+LRR domain
pGR107-PsXEG1-F	CCATCGATATGAAGGGATTCTTCGCCG	cloning full length XEG1 in *Phytophtohra sojae*

pGR107-PsXEG1-R	TCCCCCGGGGTTGACCGCAGCCGAGAAC	
pGR107-ps119627-F	GCACCAGCTAGCATCATGAAGGTTGCGTTCGCTACTG	cloning full length XEG1 homolgue Ps119627 in *Phytophtohra sojae*
pGR107-ps119627-R	CTCTAGAGGATCCCCGACGCGACGAACACTGCGG	
pGR107-ps138787-F	GCACCAGCTAGCATCATGAAGGTTCTGTTCGCCAC	cloning full length XEG1 homolgue Ps138787 in *Phytophtohra sojae*
pGR107-ps138787-R	CTCTAGAGGATCCCCCTCGCGACGAACGCGACGGA	
pGR107-PPTG_16272-F	CCATCGATATGAAGCTTTCCTTCGC	cloning full length PPTG_16272 in *Phytophtohra parasitica*
pGR107-PPTG_16272-R	TCCCCCGGGAACTGTGTGGACAGCGGCC	
pGR107-PITG_06962-F	TCAGCACCAGCTAGCATCGATATGAAGGTGTTCTTCGCTGCTG	cloning full length PITG_06962 in *Phytophtohra infestans*
pGR107-PITG_06962-R	AATCTCTAGAGGATCCCCGGGTCGTCCCCTGAAGAAGTCGG	
pGR107-VDAG_07406-F	TCAGCACCAGCTAGCATCGATATGAAGTTCATCACAGTCCTTAC	cloning full length VDAG_07406 in *Verticilium dalia*
pGR107-VDAG_07406-R	AATCTCTAGAGGATCCCCGGGACTGCACTGGGAGTACCACTG	
pGR107-NPP1-F	CCATCGATATGAACGTCCTTACATTCCTC	cloning full length NPP1 in *Phytophtohra parasitica*
pGR107-NPP1-R	TCCCCCGGGAGCGTAGTAAGCGTTGCCT	
NbCYP71D20-RT-F	CCGCACCATGTCCTTAGAG	Antje Heese et al., 2007
NbCYP71D20-RT-R	CTTGCCCCTTGAGTACTTGC	
NbEF1α-RT-F	GTATGCCTGGGTGCTTGAC	Antje Heese et al., 2007
NbEF1α-RT-R	ACAGGGACAGTTCCAATACCA	
NbSOBIR1-RT-F	CTTAGAAAAACTCTCTTTAGC	Liebrand et al., 2012
NbSOBIR1-RT-R	TATGGATTGGAGTGACATTATG	
NbSERK3-RT-F	ATTGCTGGAGGAGTTGCTGCCGG	RT check expression of BAK1 homologues
NbSERK3-RT-R	CCACGTACAGCAGTGGTAACAT	
PAR-F	ATGAACTTCCGCGCTCTGTT	*Phytophtohra parasitica* biomass analysis
PAR-R	CAGTGACGCGCACGTAGAC	

References

1. Fischer, I., Dievart, A., Droc, G., Dufayard, J.-F. & Chantret, N. Evolutionary dynamics of the leucine-rich repeat receptor-like kinase (LRR-RLK) subfamily in Angiosperms. *Plant Physiology* **170**, 1595-1610 (2016).
2. Shiu, S.H. et al. Comparative analysis of the receptor-like kinase family in Arabidopsis and rice. *Plant Cell* **16**, 1220-1234 (2004).
3. Jehle, A.K. et al. The receptor-Like protein ReMAX of Arabidopsis detects the microbe-associated molecular pattern eMax from *Xanthomonas*. *Plant Cell* **25**, 2330-2340 (2013).
4. Zipfel, C. et al. Perception of the bacterial PAMP EF-Tu by the receptor EFR restricts *Agrobacterium*-mediated transformation. *Cell* **125**, 749-760 (2006).
5. Jones, D.A. & Jones, J.D.G. The role of leucine-rich repeat proteins in plant defences. Advances in Botanical Research Incorporating Advances in Plant Pathology, **24**, (eds. Andrews, J.H. & Tommerup, I.C.) 89-167 (1997).
6. Fritz-Laylin, L.K., Krishnamurthy, N., Tor, M., Sjolander, K.V. & Jones, J.D.G. Phylogenomic analysis of the receptor-like proteins of rice and Arabidopsis. *Plant Physiology* **138**, 611-623 (2005).
7. Petre, B., Hacquard, S., Duplessis, S. & Rouhier, N. Genome analysis of poplar LRR-RLP gene clusters reveals RISP, a defense-related gene coding a candidate endogenous peptide elicitor. *Frontiers in Plant Science* **5**, 111 (2014).
