# Supplementary material for: Leucine-rich repeat receptor-like gene screen reveals that Nicotiana RXEG1 regulates glycoside hydrolase 12 MAMP detection
Source: Nat Commun. 2018 Feb 9;9:594. doi: 10.1038/s41467-018-03010-8 (PMC5807360; doi:10.1038/s41467-018-03010-8)
Supplement: Supplementary file 2 — Descriptions of Additional Supplementary Files [file 41467_2018_3010_MOESM2_ESM.pdf]

Description of Additional Supplementary Files

File Name: Supplementary Data 1
Description: Inventory of potential LRR receptor-like genes identified in *N. benthamiana* and relative TRV constructs as well as primers. 1 +, predicted with signal peptide; -, predicted no signal peptide. 2 The silencing efficiency was labeled once the corresponding construct-treated *Nicotiana benthamiana* plants were analyzed. 3The sequences were labeled as 'Correct' or 'Corrected' accroding to the transcriptom data or 'Not determined' if no transcriptom data available.

File Name: Supplementary Data 2
Description: Total number of LRR-RLKs in *N. benthamiana* and classified subgroups.1 Arabidopsis RLKs used for phylogeny were put next to the *Nicotiana benthamiana* RLKs fall into the same subgroup. 2+, predicted with signal peptide; -, predicted no signal peptide; 3RD, the kinase domain has a conserved arginine-aspartate (RD) motif in the catalytic loop; non-RD, different types of substitutions or deletion (--).

File Name: Supplementary Data 3
Description: Coding sequences of RXEG1 and RXEGL1/2 and the fragments used for silencing.
